# Supplementary material for: An integrated genomic framework for Aeromonas genomic species delineation using average nucleotide identity, core-genome phylogeny and digital DNA–DNA hybridisation
Source: Microb Genom. 2026 Sep 17;12(9):001833. doi: 10.1099/mgen.0.001833 (PMC13585408; doi:10.1099/mgen.0.001833)
Supplement: Supplementary file 1 [file mgen-12-01833-s001.pdf]

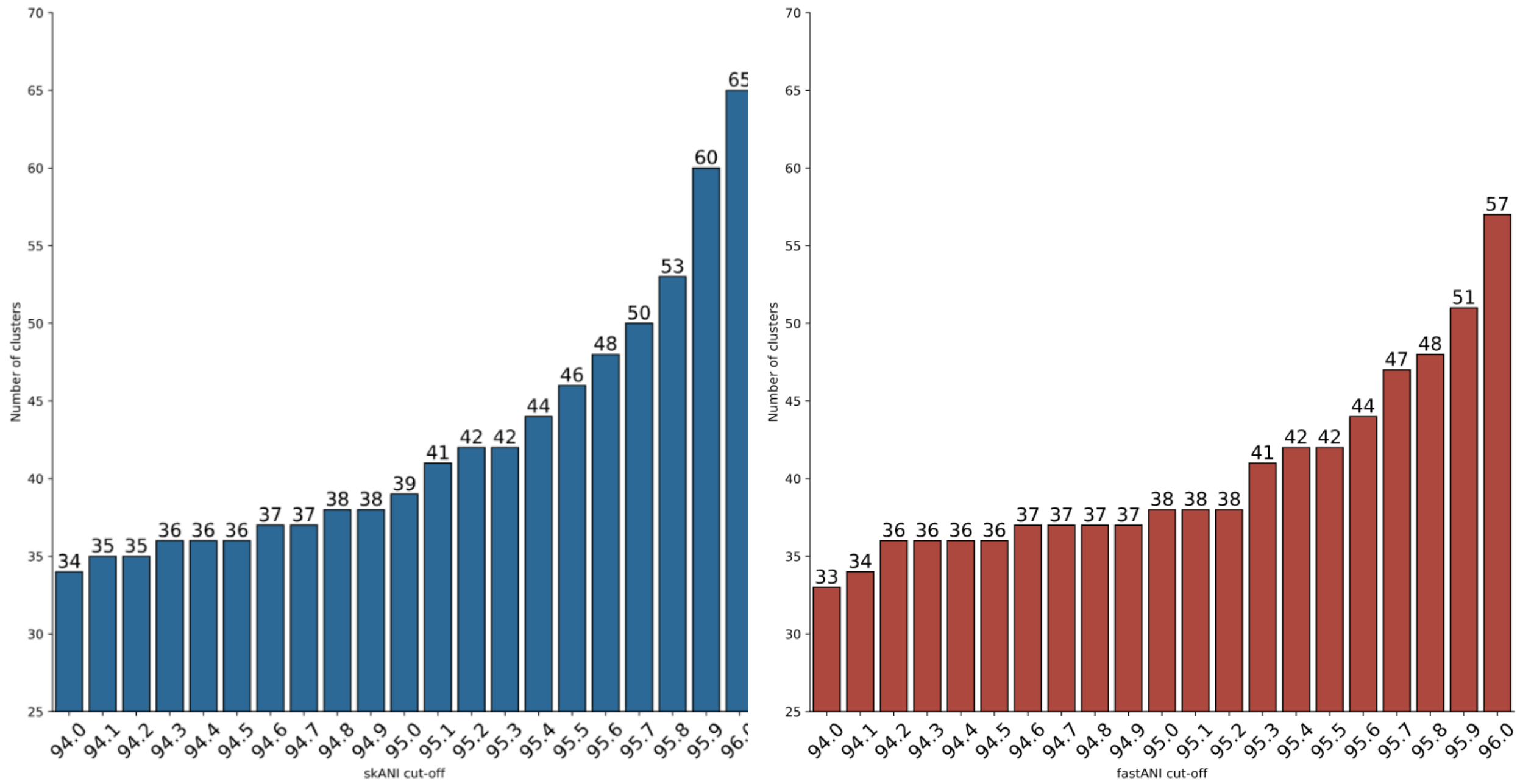

Supplementary Figure 1. Column graph representation of skANI clusters and fastANI clusters delineated at 0.1% increments

ANI pairwise comparison values generated using skANI (left, blue) and fastANI (right, red). The genomes were then delineated in clusters at 0.1% increments using average linkage clustering. These column graphs are supplementary to the Figure 1, showing cluster numbers.

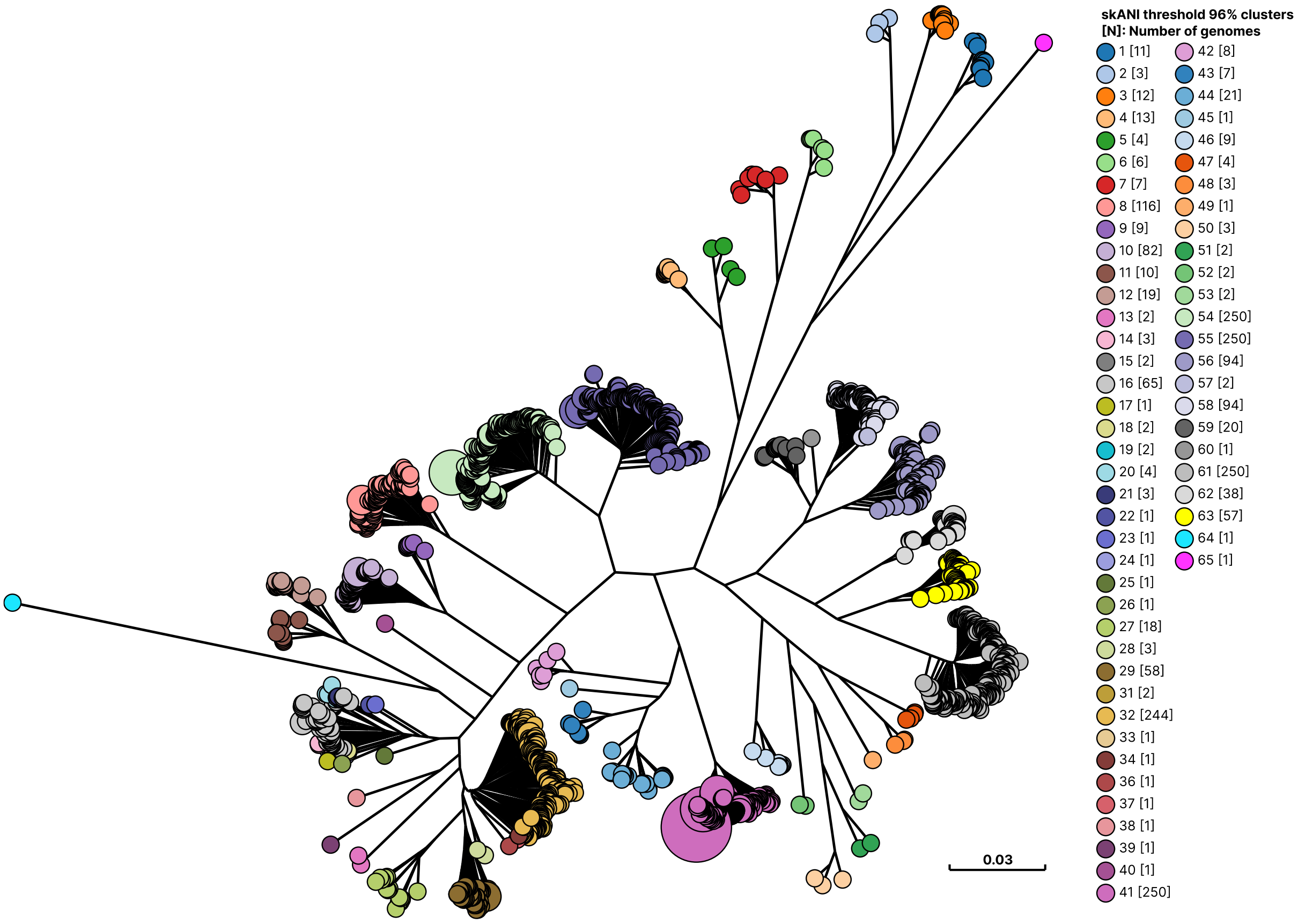

Supplementary Figure 2. Core genome phylogenetic tree representative of 4,366 genome dataset overlaid with 96% average nucleotide identity clusters (skANI)

The core genome phylogeny tree with a total of 2,083 genomes, representing the 4,366-genome dataset. The tree was overlaid with clusters at a 96% ANI cutoff using skANI. The maximum likelihood tree was constructed using concatenated alignments of 673 *Aeromonas* genus core genes as input for IQ-TREE v3.0.1 and visualized with GrapeTree v1.5.0. Not all additional clusters at 96% ANI were represented in the core genome tree. However, the additional clusters at 96% ANI do not align with the phylogenetic clades. Multiple species were separated including *A. veronii*, *A. allosaccharophila*, *A. media*, and *A. rivipollensis*,

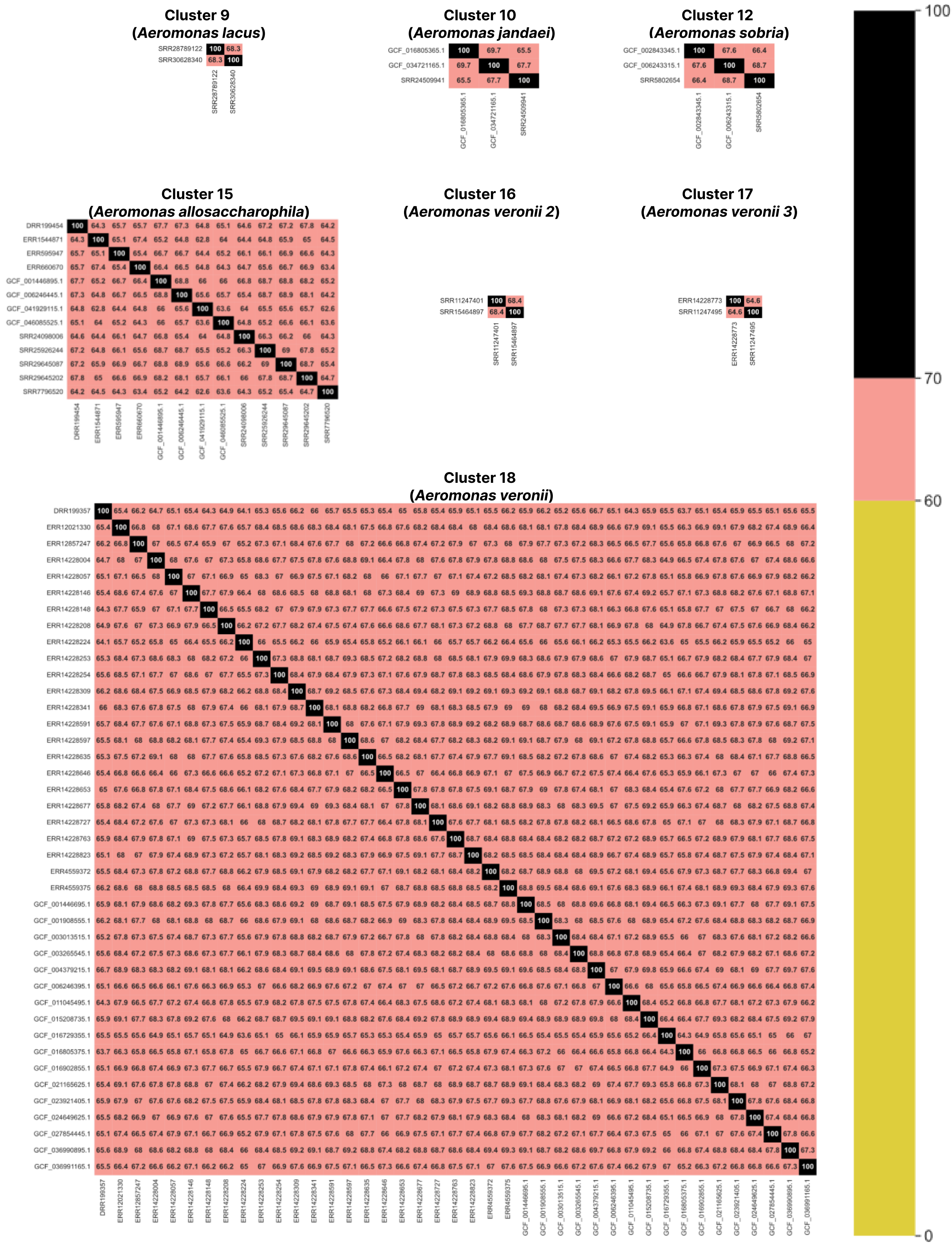

Cluster 9
(Aeromonas lacus)
SRR28789122
SRR30628340
100
68.3
68.3
100
Cluster 10
(Aeromonas jandaei)
GCF_016805365.1
GCF_034721165.1
SRR24509941
100
69.7
65.5
69.7
100
67.7
65.5
67.7
100
Cluster 12
(Aeromonas sobria)
GCF_002843345.1
GCF_006243315.1
SRR5802654
100
67.6
66.4
67.6
100
68.7
66.4
68.7
100
Cluster 15
(Aeromonas allosaccharophila)
DRR199454
ERR1544871
ERR595947
ERR660670
GCF_001446895.1
GCF_006246445.1
GCF_041929115.1
GCF_046085525.1
SRR24098006
SRR25926244
SRR29645087
SRR29645202
SRR7796520
Cluster 16
(Aeromonas veronii 2)
SRR11247401
SRR15464897
100
68.4
68.4
100
Cluster 17
(Aeromonas veronii 3)
ERR14228773
ERR11247495
100
64.6
64.6
100
Cluster 18
(Aeromonas veronii)
DRR199357
ERR12021330
ERR12857247
ERR14228004
ERR14228057
ERR14228146
ERR14228148
ERR14228208
ERR14228224
ERR14228253
ERR14228254
ERR14228309
ERR14228341
ERR14228591
ERR14228597
ERR14228635
ERR14228646
ERR14228653
ERR14228677
ERR14228727
ERR14228763
ERR14228823
ERR4559372
ERR4559375
GCF_001446695.1
GCF_001908555.1
GCF_003013515.1
GCF_003265545.1
GCF_004379215.1
GCF_006246395.1
GCF_011045495.1
GCF_015208735.1
GCF_016729355.1
GCF_016805375.1
GCF_016902855.1
GCF_021165625.1
GCF_023921405.1
GCF_024649625.1
GCF_027854445.1
GCF_036990895.1
GCF_036991165.1
100
70
60
0

Supplementary Figure 3. Intraspecies dDDH values below 70% observed in seven clusters delineated by skANI threshold of 95.4%

Further dDDH comparisons were made within ANI clusters delineated at skANI threshold of 95.4% (fastANI threshold of 95.6%). It was found that seven clusters, five corresponding to existing taxonomic species, have genomes with pairwise dDDH values below 70%, but above 60%, which are presented as heatmaps.

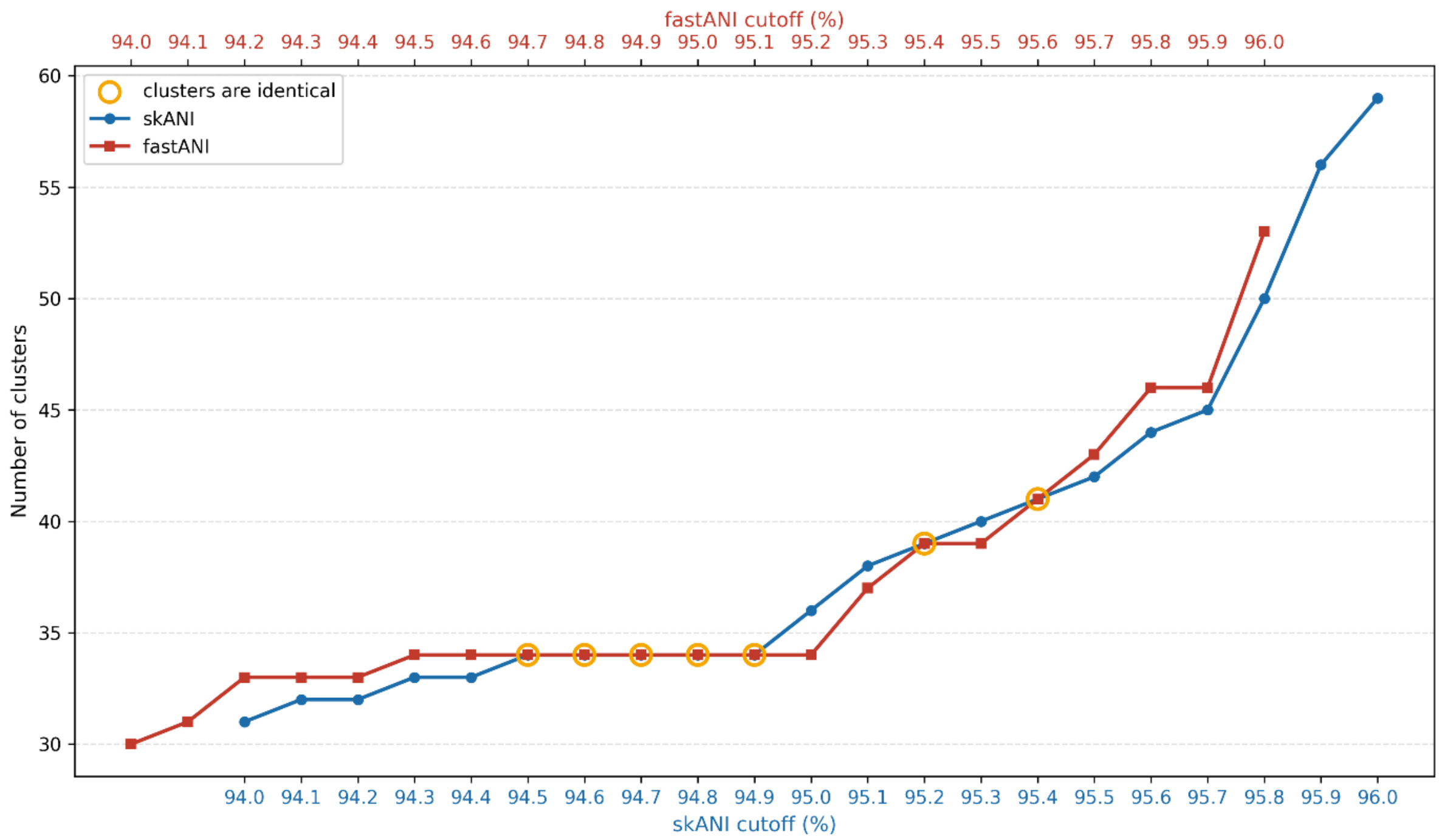

Supplementary Figure 4. Number of genome clusters delineated from the initial 3,782 *Aeromonas* genomes at different average nucleotide identity (ANI) cutoffs generated from skANI and fastANI with a 0.2% offset

ANI average linkage of 3,782 *Aeromonas* genomes using fastANI and skANI. The clusters between 94.5% to 95.4% skANI cutoffs match for most values between 94.7% to 95.6% fastANI cutoffs. The x-axis shows ANI cutoffs ranging from 94% to 96% for skANI (blue), with a 0.2% offset for fastANI (red). The y-axis shows the number of ANI clusters. Cutoffs where the number of clusters and genomes assigned to each cluster are identical between the two tools are demarcated with a yellow circle.

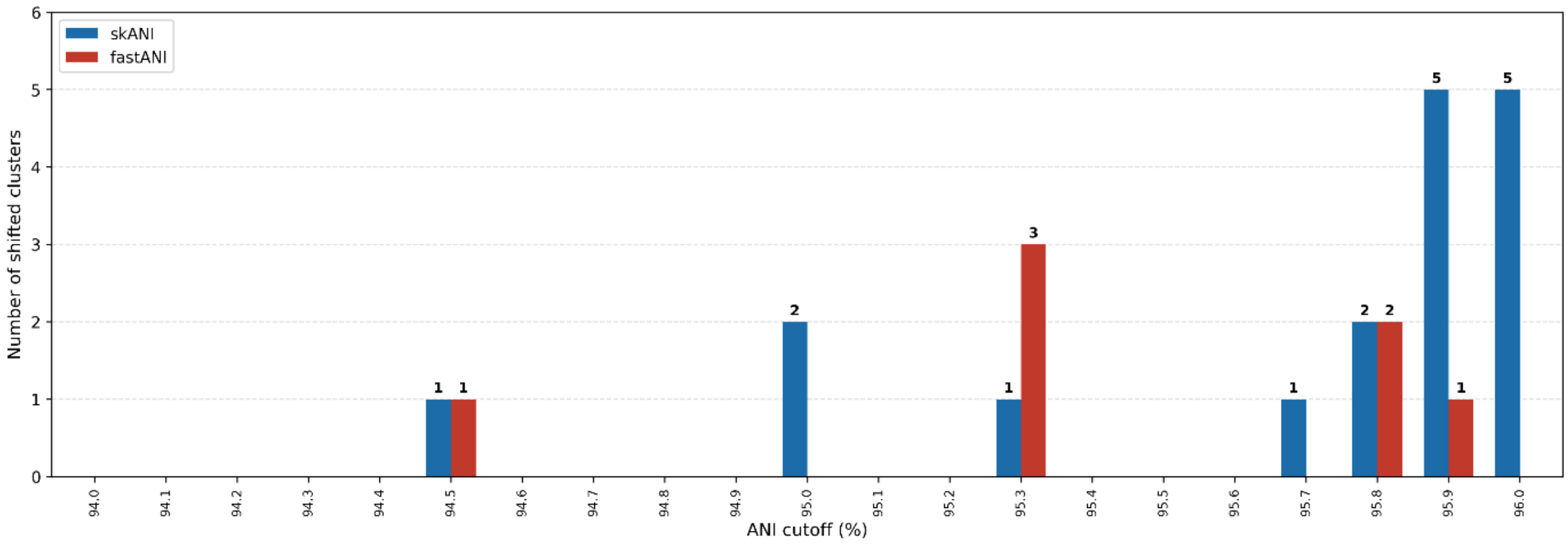

Supplementary Figure 5. Number of cluster shifts observed between the shared 3,782 genomes of the two datasets

Comparison of the cluster assignments of the 3,782 genomes between the two datasets showed that at the selected threshold of 95.4% skANI (95.6% fastANI), cluster assignments were completely identical between the datasets, demonstrating that the selected threshold is robust to dataset expansion (see Supplementary Table 2).

Across the range of ANI thresholds evaluated, the same genomic clusters were recovered in both datasets. For some clusters, the ANI threshold at which they became delineated differed by only 0.1%, likely reflecting the addition of new genomes in the expended dataset (Supplementary Figure 3). Importantly, these minor shifts did not affect the selection of the operational threshold of 95.4% skANI (95.6% fastANI).
